# Factors and misperceptions of routine childhood immunization service uptake in Ethiopia: findings from a nationwide qualitative study

**DOI:** 10.11604/pamj.2017.28.290.14133

**Published:** 2017-12-05

**Authors:** Tefera Tadesse, Kinde Getachew, Tersit Assefa, Yohannes Ababu, Tesfaye Simireta, Zewdie Birhanu, Yohannes Hailemichael

**Affiliations:** 1 Jimma University, Jimma, Ethiopia; 2 Immunization Program, UNICEF, Addis Ababa, Ethiopia; 3 Immunization Program, World Health Organization, Addis Ababa, Ethiopia

**Keywords:** Childhood immunization, Ethiopia, factors, qualitative study

## Abstract

**Introduction:**

While the routine childhood immunization program might be affected by several factors, its identification using qualitative evidence of caretakers is generally minimal. This article explores the various factors and misperceptions of routine childhood immunization service uptake in Ethiopia and provides possible recommendations to mitigate them.

**Methods:**

In this study, we used a qualitative multiple case study design collecting primary data from 63 focus group discussions (FGDs) conducted with a purposefully selected sample of children's caretakers (n = 630).

**Results:**

According to the results of this study, the use of routine childhood immunization is dependent on four major factors: caretakers' behavior, family characteristics, information and communication and immunization service system. In addition, the participants had some misperceptions about routine childhood immunization. For example, immunization should be taken when the child gets sick and a single dose vaccine is enough for a child. These factors and misperceptions are complex and sometimes context-specific and vary between categories of caretakers.

**Conclusion:**

Our interpretations suggest that no single factor affects immunization service uptake alone in a unique way. Rather, it is the synergy among the factors that has a collective influence on the childhood immunization system. Therefore, intervention efforts should target these multiple factors simultaneously. Importantly, this study recommends improving the quality of existing childhood immunization services and building awareness among caretakers as crucial components.

## Introduction

Immunization program is one of the primary strategies for achieving the Sustainable Development Goals (SDGs) [1]. However, it remains an unfinished business as dozens of studies reveal millions of children worldwide have not yet benefited from the protection vaccines are supposed to provide [2, 3]. In Ethiopia, the Expanded Program on Immunization (EPI) which was initiated in 1980 has a goal of protecting children against the eight vaccine preventable diseases, namely tuberculosis, measles, poliomyelitis, tetanus, diphtheria, whopping cough, hepatitis-B and H-influenza [4]. More recently pneumococcal vaccine has been introduced and currently, Ethiopia is providing 10 antigens targeting major killer diseases during childhood [5]. However, empirical evidence show that the target goal of EPI has not been achieved, so far in Ethiopia [6]. As the Ethiopian Health Sector Transformation Plan (HSTP) document indicates, EPI is expected to cover 100% of the target population, however, the coverage survey report of 2009 shows that 79% of the target was achieved. A recent national survey also reveals comparable results to previous findings in which DPT-3 vaccine and measels vaccine coverage was 78% and 68% respectively [7]. Moreover, the same source highlights the presence of significant regional variations in vaccine coverage. Existing studies of child immunization in the developing countries are primarily quantitative with a focus on the magnitude and completion rates, than others [4, 8-12]. Moreover, while immunization of children might be affected by several factors, its identification using qualitative evidence collected from children's caretakers is generally minimal and narrow addressing aspects of the possible factors, often based on global reviews [13-17]. Although global reviews, for example, Rainey et al (2011), can play a useful role in identifying key questions, local enquiry and follow-up remain essential in providing concrete evidence about the existing realities and context specific factors. Unlike to the quantitative investigations, the qualitative investigations, however, provide detailed views of caretakers in their own words, complex analyses of multiple perspectives and specific contexts of different locations that shape caretaker experiences with routine childhood immunization [18]. Moreover, qualitative inquiry offers the opportunity to involve caretakers and this can enhance the validity of caretaker views uncontaminated by others perspectives [19]. On top of this, existing research on the topic of immunization is fragmented, addressing issues surrounding either fully vaccinated, unvaccinated or dropout in specific localities [20]. Hence, the purpose of this study was to identify the factors influencing childhood immunization, using a qualitative research approach. More specifically, this study addresses the following research questions; 1) What does childhood immunization practice seem in Ethiopia based on the caretakers' responses? 2) What are the factors and misperceptions associated with routine childhood immunization in Ethiopia based on the caretakers views and perspectives? 3.To what extent do caretakers'? responses on the factors and misperceived benefits of routine childhood immunization have similarities and differences across the different categories and administrative regions?

## Methods

**Figure 1:**
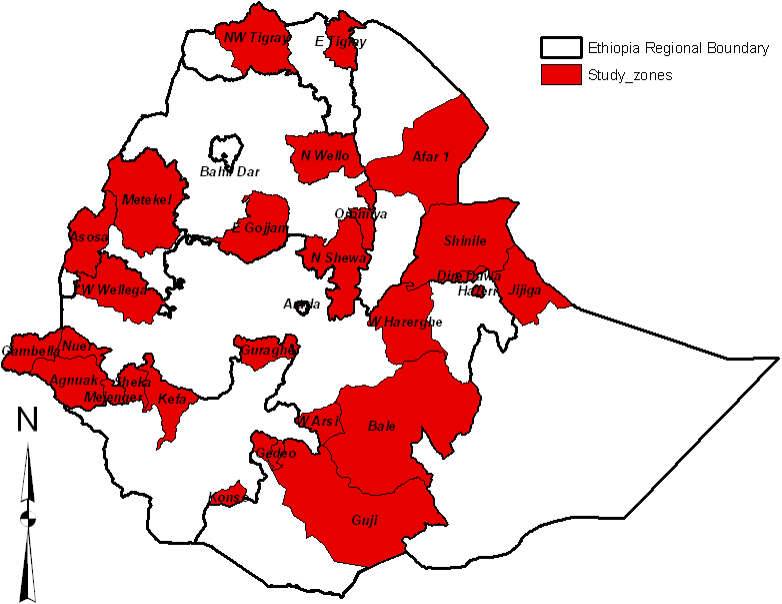
The administrative regions of the study area

The study was conducted in all the nine regional states and two city administrations in Ethiopia via employing a qualitative case study design. This design has several advantages for this type of investigation allowing the researchers to build a holistic, detailed description and analysis of the factors associated with routine childhood immunization within its real world context [21]. The study population consisted of caretakers who had children aged 12-23 months during the time of data collection. In total, 63 focus group discussions (FGDs) were conducted Figure 1. Study participants were caretakers clustered into two: those whose children were vaccinated and those whose children were not vaccinated or dropout of the immunization schedule. Caretakers of immunized children have been either completed or have the last appointment schedule to immunize their children sometime in the future. Table 1 presents a summary of the distribution of FGD participants across categories and administrative regions. We used the FGD guideline that addresses issues such as the demographic information of caretakers and factors possibly affect the use of routine child immunization services. The FGD data were collected by professionals holding Master's in public health /social sciences. The overall data collection was supervised by the principal investigators (PI) and co-principal investigators (co-PIs).The study was approved by Jimma University's ethical review board and the National Ethical Review Board of Ministry of Science and Technology in Ethiopia (Reference No: 310/622/04). Permission was obtained from administrator at all levels. The aim of the study was explained to the respondents and an informed verbal consent was obtained from each respondent. All FGDs were transcribed verbatim and translated from local languages into English. The transcribed data were reviewed and commented by the researchers. The data collectors received timely comments on the transcribed data. The transcribed data were carefully read by the investigators. After several reviews, key categories and themes were identified based on the objectives of the study. In this study, we used thematic analysis, incorporating an explanation of elements explored in-depth [22]. Data analysis was ongoing during the research process and allowed the researchers to condense an extensive amount of information into a more manageable format [23]. Analysis involves organizing data, breaking them into more manageable parts, developing codes and searching for possible patterns for a comparative perspective. In order to organize the data, we used comparison table-used to compare views of groups of caretakers on one theme and demographic table of participant numbers across the administrative regions included in the research site [24].

**Table 1:** The distribution of caretakers FGD participants by category and administrative region

Region	Caretakers whose children were immunized	Caretakers whose children were unimmunized or dropout
	No of FGDs	No of FGDs
Addis Ababa	2	2
Afar	2	4
Amhara	4	4
Benishangul Gumuze	4	-
Dire Dawa	2	2
Gambella	4	5
Harari	4	-
Oromia	5	4
Somali	2	2
Southern Nations, Nationalities, and People	4	2
Tigray	2	2
Total	36	27

## Results

In the final analysis, three working themes were generated. These themes include the status of routine childhood immunization; factors associated with routine childhood immunization and perceived misperceptions of routine childhood immunization. Each factor identified has a further breakdown of sub-factors. Tthe main findings of the study regarding these emerging themes are presented in Table 2, Table 3 .

**Table 2:** Lists of factors affecting routine childhood immunization in Ethiopia across category and administrative regions

Factor	Responses of caretakers of immunized children											Responses of caretakers of unimmunized or dropout children										
**Immunization service system**	1	2	3	4	5	6	7	8	9	10	11	1	2	3	4	5	6	7	8	9	10	11
Inaccessible health facility		×	x		x	x	x			x			x	x		x				x		
Lack of immunization service	x							×	x													
Health workers motivation, attitude and competence		x							x													
Resources/logistics													x			x						
False Contraindications																						
Reliability of immunization services													x									
Inconvenient immunization time			x			x																
Lengthy waiting time				x	x								x	x			x		x	x	x	
**Information communication & health education**																						
Information or health education during scheduled vaccination day	x	x	x	x	x	x	x		x		x		x	x		x	x		x	x	x	
Poor announcement (Particularly for campaign)		x			x		x						x	x				x	x	x		
**Family characteristics**																						
Income/socioeconomic status		x											x									
Mobility of the pastoral community		x							x				x							x		
Parental/Husband’s support		x			x								x	x	x	x	x	x	x		x	x
**Caretaker’s behavior (knowledge, attitudes, belief & practice)**																						
Awareness on childhood immunization	x	x	x	x	x	x	x	x	x	x	x		x	x	x	x	x		x	x	x	x
Trust/Efficacy on vaccination		×			x	x		x	x													
Fear of side effects			x	x		x	x			x			x	x		x						
Fear of injections				×		x			x													
Competing priorities (Busy to win daily bread)						x	x						x	x		x				x		
Religious/cultural/beliefs/norms and rumors			×			x	x				x		x					x				
Perceived importance of vaccination for child's health			x			x	x	×		x				x			x	x				
Illness of caretaker or eligible child						x				x			x	x		x				x		
Loss of immunization card							x		x													
Forgetting the appointed date						x			x							x						

Note: ^1^Addis Ababa, ^2^Afar, ^3^Amhara, ^4^Benishangul Gmuze, ^5^Dire Dawa, ^6^Gambella, ^7^Harari, ^8^Oromiya, ^9^Somali, ^10^Southern Nations, Nationalities, and People, and ^11^Tigray

**Table 3:** List of caretakers’ misperceived benefits of childhood immunization across administrative regions studied

Misperceived benefits of immunization	Responses of caretakers of immunized, unimmunized and dropout children										
	1	2	3	4	5	6	7	8	9	10	11
Vaccination protects children from any health problem or it resolves any health problem		*√*		*√*	*√*	*√*	*√*		*√*	*√*	
Immunization prevents a child from fever, flu, malaria, diarrheal disease, and headache	*√*	*√*		*√*	*√*	*√*		*√*			*√*
The vaccine protects children from Tracoma			*√*						*√*		
Immunization prevents cholera	*√*	*√*		*√*	*√*						*√*
Vaccination helps to improve appetite						*√*					
Immunization prevents against HIV/AIDS								*√*			
Considering immunization as a treatment when the child gets sick		*√*		*√*	*√*				*√*	*√*	
Considering a single dose vaccine is enough		*√*		*√*	*√*				*√*		
Immunization should be taken every month										*√*	

Note: ^1^Addis Ababa, ^2^Afar, ^3^Amhara, ^4^Benishangul Gumuze, ^5^Dire Dawa, ^6^Gambella, ^7^Harari, ^8^Oromiya, ^9^Somali, ^10^Southern Nations, Nationalities, and People, and ^11^Tigray

**The status of routine childhood immunization**: The results reveal that non-immunization and dropout are commonly found across the different regions. The presence of these groups in almost every region suggests that childhood immunization and dropout are shared traits across the different administrative regions. Regarding their views, all caretakers of immunized children have the opinion that all or most children in their community have got immunized or they felt that no child was left unimmunized in their respective communities. Contrary to this, however, those caretakers of unimmunized children across the different regions had the opinion that few children were fully vaccinated in their respective communities. They also explained that the majority of children in their respective communities at least started vaccination so the problem was more of discontinuity or defaulting.

**Factors affecting routine childhood immunization**: Regarding the factors, the responses of the caretakers were categorized based on their child's immunization status as well as the location of their administrative regions. As per the results of this study, the main factors associated to childhood immunization include immunization services, information and communication, family characteristics and caretaker's knowledge, attitudes, beliefs and practices. Interestingly, each factor has a number of sub-factors. These key factors of childhood immunization (not in order of importance) are shown in Table 2. As shown in Table 2, the most commonly shared factors among the discussants were inaccessible health facility, lack of immunization service, poor motivation, unfavorable attitude and incompetence and bad treatment of health workers (HWs). Likewise, the other shared factors include lack of resources/logistics, restricted vaccine open policy, inconvenient immunization time, lack of information at times of vaccination day and prolonged waiting time. The various caretakers said that immunization service utilization in the outreach and house-to-house visits are not adequate (Oromia). Likewise, other discussants reported that HWs lack of commitment to help the community and mistreatment of clients by HWs are the reasons for people's dissatisfaction with the immunization services (Afar, Gambela, & Somali). However, other discussants in most administrative regions highlighted that the inability of HWs to provide information on childhood immunization during the services which was ascribed to inadequate communication skills and lack of willingness. Besides, some discussants said that failure to provide the service for children who lost immunization card hampered some caretakers from getting the service (Afar, Oromia & Somali). Mishandling, poor reception, and disrespect of caretakers from the HWs are common during immunization in some of the regions, though some participants acknowledged good reception and services of the HWs.

One of the participant caretakers expressed her concerns as follows: the health professionals didn't welcome the clients properly rather they argue with us and make an unacceptable dialogue. In order to avoid such types of arguments and unethical dialogue, we missed the rest of immunization schedules. The HWs didn't understand our problems. (A twenty-seven year-old defaulted mother from Afar). In support of the preceding view, another participant from Oromia region whose child is fully immunized commented on the mistreatment of HWs as follows: the HWs let you down, they did not respect you! They use to say why you don't keep the card just like your child! But, sometimes we lost the card! That is not deliberate! But they never accept you without the vaccination card. So, when one observes while health workers disgrace a mother who does not have a card, she will never go for vaccination if she lost the card too because she feels she will experience the same thing. So, people say “why do I receive such embarrassment while my child is healthy!” As a result, they prefer to discontinue the vaccination program. With respect to information and communication, HWs were the main source of information about immunization though the info provided from these sources lacks consistency across the administrative areas. In some administrative regions like Addis Ababa and Tigray, health care providers were found to be good sources of information and communication. However, some FGD participants in Gambella, Somali, Afar and Benishangul Gumuz, said that they have got a little information on immunization during a visit for the services. One of the participating mothers (a thirty-nine-year-old mother from Afar whose child was dropped out) said that: “*no one provided us information about the vaccines advantages and disadvantages. The HWs didn't inform us about the benefits of immunizations. They simply told us the next appointment*.” Similarly, a participant in Benishangul Gumuz said that “*HWs sometimes provide information. But most of the time they simply administer the vaccine*". One of the participating mothers from Gambella said: “*They simply administered the vaccine and told me when to come back*.” A twenty-eight year-old mother from Somali said: “*The health providers administered injections to our children and told us only the next immunization day. No further information was given to us*.” This verifies the presence of differences and heterogeneity in the way HWs provide information for their clients. Family demographic characteristics like socioeconomic status and mobility of pastoral community were strong barriers in some administrative regions. A defaulted participant from Afar region, said that “*my child discontinued due to a personal problem of being busy with my household chores and got no time to take my child to HC on schedules*.” Also, a twenty-five year-old mother from Dire Dawa did not start an immunization for her child because she gave birth in a remote distant place where immunization service was not available.

Another defaulted mother from Somali cited personal reasons of sickness and busy routine schedule. Moreover, long distance is a particular problem for discontinuity and refusal for the rural women and for those who lived in the far distance of the health facilities. In terms of awareness, most of the caretakers of immunized children were found aware of the basic knowledge of childhood immunization compared to the large majority of caretakers with unimmunized and dropout children. However, this does not mean that all the caretakers in each category are generally knowledgeable or otherwise. Hence, there is a possibility of having immunized or non-immunized or dropout children, regardless of being aware or unaware of childhood immunization. Most caretakers (vaccinated mothers, unvaccinated mothers) confirmed that mothers are responsible to immunize children. Favoring the approval of the mother, a mother from AA, argued: “*Usually it is a mother's duty and responsibility to bring the child to a health facility for immunization*". However, caretakers who did not immunize their children in Afar region and Dire Dawa argued that fathers as well play a central part in deciding on child immunization. A defaulted mother from Afar added that “*the very reason for the importance of the father's decision is mainly because the mothers cannot read and write so that the father or the husband is the one who is responsible to facilitate and support*". Lack of awareness was found the most common factor perceived to affect routine childhood immunization in Ethiopia. A deeper analysis of the responses of the two groups (Caretakers' of Immunized Children and those of Unimmunized or dropout Children) reveals larger similarities and few differences regarding their views and perspectives. For example, those caretakers whose children have been immunized perceived the side effects of immunization as a temporary health problem. However, those caretakers whose children have been unimmunized or dropped out recognized those side effects as a serious health problem. A mother whose child is not vaccinated perceived the same problem as a serious health issue and attributed the side effect to wrong injection or overdosing of the vaccine. For instance, a mother whose child was not vaccinated said, “*Vaccine can have some side effects due to overdosing on the drug or if injected wrongly*” (Dire Dawa). Also, the other caretaker whose child was defaulted for measles due to experiencing some side effects which led to further fear and failure said: my child was seriously sick when she took the vaccination. It was hard to continue that way for the next schedule since the illness was very serious leading her to a serious leg spasm and high fever, hence my husband and I discussed that and decided to quit it (A 25-year- old defaulted mother, from Dire Dawa). Analysis of caretakers' view across the different administrative regions of the country revealed some general differences. Factors equally important in all regions were lack of awareness. Also, common factors in some regions include lack of trust about the efficacy of vaccination, fear of side effects and injections. Besides, other factors commonly found in some regions include religious/cultural/beliefs, norms and rumors, illness of caretaker or eligible child, forgetting the appointed date, loss of immunization card and competing priorities. It is noteworthy that many of the major factors are quite consistent across administrative regions, although a few stand out in particular locations.

**Misperceived benefits of immunization**: The FGDs analysis revealed that there were significant misperceived benefits of immunization in the community. Table 3 shows common misperceived benefits of immunization across the respondents' administrative regions. As shown in Table 3, there are different misperceptions associated with routine childhood immunization across administrative regions. The misperception is presented across only administrative regions since there was no clear pattern of difference between the categories of sampled caretakers whose children were immunized and unimmunized or dropout. From the practical perspective, these factors can be clustered into two major categories: positive factors and negative factors. For instance, considering immunization as a protection or a cure for any health problem or a range of health issues have a positive message for caretakers encouraging them to immunize their kids. Besides, it looks positive to have a belief that immunization prevents from HIV and AIDS or have the power to amend the child's appetite so that the kids eat well. However, the feelings that immunization should be taken when the child gets sick or a single dose vaccine is enough has a negative rhetoric potentially obstructing caretakers to reap the benefits of full immunization. A cursory look at the regional distributions of the caretakers' misperceptions across the different regions reveal that the misperceptions were commonly shared across the different regional states and city-administrations involved.

## Discussion

This study explored the factors and misperceptions of routine childhood immunization uptake through a qualitative multiple case study approach. The main factors of non-immunization or dropouts were related to immunization service provisions and to caretakers' psychosocial factors. Evidence from the 20^11^ national demographic and health survey and the 20^12^ national survey in Ethiopia identified a number of behavioural determinants of full immunization coverage testifying the multiple factors contributing to full immunization of children in Ethiopia [7, 25]. Also, it was clear from the same data that aspects of caretakers' demographic characteristics were significantly positively related to their immunization practice for the sample group studied. Likewise, another study conducted at Wonago district revealed a number of negative factors associated with non-immunization and dropout of childhood immunization [15]. This study, using FGD with caretakers obtained almost similar results. As the result shows, childhood immunization was affected by several complicated factors, often involving a combination of interacting factors. This result was consistent with earlier findings [10, 15, 18]. Understandably, certain factors put children at risk of missing immunization, for example, place of residence, family income-but it is the interaction of multiple factors, in a very personal way, that lead to a particular family's decision to have its children fully immunized or not [9, 17]. This is also true in the current study exemplifying the diversity of factors associated with childhood immunization services. In some cases of incomplete immunization, the explanation may have one simple factor accounting for. For example, the father prohibited the caretaker to return after the child experienced a fever following immunization (Afar), or the caretaker cannot be away from work during immunization hours (Dire Dawa, Harar & Oromia). In other instances, the cases might be a combination of beliefs, perceptions, knowledge and experiences (Afar & Somali). A child should not be denied immunization service, though immunization card is not available. The HWs should review the child's records and give the necessary vaccine accordingly. It is advisable to provide a new card to caretakers who lost their cards to facilitate and encourage next visit. For immunization of children's coverage to improve sustainability in Ethiopia, investments and efforts will be required in multiple areas targeting caretakers regardless of their children immunization status and location in some form and yet it will need to be tailored to the specific needs of caretaker groups and individual regions [26].

**Limitations**: While, the categories, factors and misperceptions are used to explain the caretakers' perceptions of the existing realities representing immunization, they cannot capture the multi-causality of un-immunization or dropout. Perhaps new measures and approaches might better describe the multiple factors and the relationship between them.

## Conclusion

This study identified various factors and misperceptions that affect routine childhood immunization uptake. The results reveal that each factor does not function in isolation; rather it is the synergy among the factors that has a collective influence on the childhood immunization system. The implication is that intervention efforts should target these multiple factors simultaneously. To this effect, improving the quality of existing childhood immunization services and building awareness among caretakers are crucial components. Nonetheless, knowledge and practice gaps seemed more prominent in some regions such as Afar, Gambella, and Somali and this need special attention. At each immunization-contact, HWs need to provide adequate information for caretakers, particularly when to return for the next immunization doses, the number of visits/doses remaining and the benefits of each vaccine in specific conditions. Moreover, they should explain about side effects of the vaccine, provide counseling for multiple injections and children crying. Likewise, it is important to tell the importance of keeping immunization card; and in conclusion, check whether they correctly understood the information provided. Caretakers should be informed that if they are busy, fathers/relatives or any other person could bring children with an immunization card to the health facility. To promote routine childhood immunization service utilization, HWs should be empathetic, build relationship and treat caretakers with dignity and respect. Likewise, they should welcome caretakers even when they lost their immunization card. Moreover, immunization program needs to be organized /reorganized to make them as convenient and acceptable to caretakers. For example, reducing long waiting time, arranging appointments on convenient days like market days; and encouraging caretakers to come back on the appointed date are among the worthwhile recommendations. The mobile immunization program is deemed right in some settings such as in pastoralist areas (Afar & Somali) to increase the proportion of immunized children. The provision of supplementary foods together with vaccination needs caution; otherwise, it discourages caretakers to come for vaccination unless it is there. Also, HWs should take advantage of other platforms to deliver basic information and facts on immunization, for example, social, religious and cultural gathering and events. Along with, HWs are required to provide home-based health education to caretakers using pictorial messages. Moreover, it is necessary that HWs counsel caretakers about misperceptions and wrongly perceived benefits of immunization. Furthermore, they should explore and address caretakers' perceptions about routine immunization and campaign programs. Moreover, it is important to provide information to caretakers on possible differences between a campaign and a routine program.

### What is known about this topic

- Previous research works on the uptake of routine childhood immunization in sub-Saharan Africa, mainly studied the barriers and challenges apparent in the health system affecting the implementation of childhood immunization;
- It is also known that uptake of immunization service is dependent on caretakers, health system and health care providers-based factors and these factors are complex and vary across low- and middle-income countries, with some factors coinciding among these country groups.

### What this study adds

- This study explored caretakers immunization service utilization considering the behavioral aspects of caretakers and inquired how those factors and misperceived benefits of routine childhood immunization hinder immunization service uptake in local context. These qualitative findings provide relevant data as basis for public health communication strategies to reduce under vaccination/dropout rate of immunization service;
- Improving caretakers immunization service utilization requires much more than the results of survey on their experiences. We also identified key barriers that affecting the characteristics and personal behaviors that are related with immunization service utilization;
- Above all, this study explored the caretaker's behavior, information and communication, family characteristics and immunization service system through a qualitative multiple case study approach. The results have a number of practical implications for Ethiopia and other health systems in sub-Saharan Africa and in particular for health institutions and programs working on childhood immunization service at the national and sub-national levels.

## Competing interests

The authors declare no competing interests.
